# Epicardial pulsed-field ablation-impact of electric field and heat distribution induced by coronary metallic stents

**DOI:** 10.3389/fcvm.2024.1445424

**Published:** 2024-08-29

**Authors:** Zhen Wang, Ming Liang, Jingyang Sun, Jie Zhang, Yunhao Li, Lisheng Xu, Yaling Han

**Affiliations:** ^1^ College of Medicine and Biological Information Engineering, Northeastern University, Shenyang, China; ^2^ Department of Cardiology, General Hospital of Northern Theater Command, Shenyang, China; ^3^ National Key Laboratory of Frigid Zone Cardiovascular Diseases, General Hospital of Northern Theater Command, Shenyang, China; ^4^ Graduate School of China Medical University, China Medical University, Shenyang, China

**Keywords:** pulsed electric field ablation technique, atrial fibrillation therapy, computer modeling, metal stent, ablation region

## Abstract

**Background:**

Pulsed-field ablation (PFA) technique is a nonthermal ablation technique. No study has yet evaluated the effect of the positional relationship between the ablation electrode (AE) and the coronary metal stent (CMS) on the electric field distribution and temperature distribution in epicardial ablation. Our study aimed to evaluate the effect of the CMS on the electric field as well as the temperature distribution in different models.

**Methods:**

Multi-angle modeling of the CMS and AE was performed. The PFA ablation region was evaluated with a field strength contour of 1,000 V/cm, which was used to assess the validity of the two-dimensional (2D) model simulation data as well as the distribution of the multi-angle electric field and temperature in the three-dimensional (3D) model.

**Results:**

The presence of the CMS had little effect on the width of the ablation area (0.2 mm). In the 3D model, the temperature of the ablation area was highest when the angle between the AE and the CMS was in the 90° position (43.4°C, 41.3°C); a change in the distance between the AE and the CMS affected the temperature of the ablation area (maximum 2.1°C) and the width of the ablation (maximum 0.32 mm).

**Conclusion:**

The presence of the CMS distorts the distribution of the electric field, but does not produce a change in the extent of the ablation damage, nor does it bring thermal damage to the ablation region. Different simulation models give similar results in PFA calculations, and this study effectively reduces the complexity of modeling simulation.

## 1 Introduction

Atrial fibrillation (AF), one of the most common clinical arrhythmias, and pulsed electric field ablation (PFA) has recently been proposed by researchers for the treatment of AF (1–6). PFA was used in the treatment of cardiac arrhythmias as early as 1980, but due to the limitations of the technology at that time, it could cause other complications. With the development of this ablation device, this ablation technique has rekindled the interest of researchers (7–10). The researchers found that compared with radiofrequency ablation and cryoablation, PFA has a better safety profile, such as reducing the chance of complications such as phrenic nerve injury and pulmonary vein stenosis during endocardial ablation (11–13). Early animal studies demonstrated that arrhythmias can be induced by stimulation of the cardiac nervous system. These nervous systems are interconnected with ganglionic plexus of the epicardium, which are usually embedded in the fatty layer of the epicardium (14–16).One study demonstrated that PFA for ablation of epicardial ganglionic plexus could effectively treat AF, and also found that the presence of ganglionic plexus distorted the normal electric field in the computational model, which may be due to the higher electrical conductivity of the ganglionic plexus compared to its surrounding tissues (17, 18). Follow-up researchers hypothesized that metal stents inside coronary arteries might have a more pronounced distorting effect on the electric field and cause a local temperature increase, and Hogens found experimentally and computationally that during electrode ablation of tumors, a distortion of the electric field distribution was observed when the metal stent was close to the ablation electrode. In another study, it was found that although the heat of the metal stent itself does not increase dramatically, it can be observed that the temperature near the ablation electrode increases due to the presence of the metal stent (19–21). In a later study, ANN et al. found that the presence of the metal stent caused a distorted distribution of the electric field of the ablation electrodes and increased the temperature of the tissues between the ablation electrodes, but this temperature change did not cause thermal damage to the nearby tissues by calculating the two-dimensional computational model. Later, it was found that in the 3D model calculation, the difference between the simplified 3D model and the real complex 3D model ablation area and the depth of the ablation area is negligible, which simplifies the complexity of 3D model modeling (22, 23). Given that it has been shown that the presence of metal brackets induces a distorted distribution of the normal electric field and that the size of the ablation region between the simplified 3D model and the real complex 3D model is negligible, it is necessary to analyze and calculate both the 3D model and the 2D model in the computational domain. To this end, we conduct a computer modeling study to evaluate the effect of the presence of the metal bracket in the 3D model and the 2D model and to analyze and compare the gap between the electric field distributions as well as the temperature distributions of the two models, to reduce the complexity of the modeling simulation calculations.

## 2 Methods

### 2.1 Modeling

**Figure 1:**
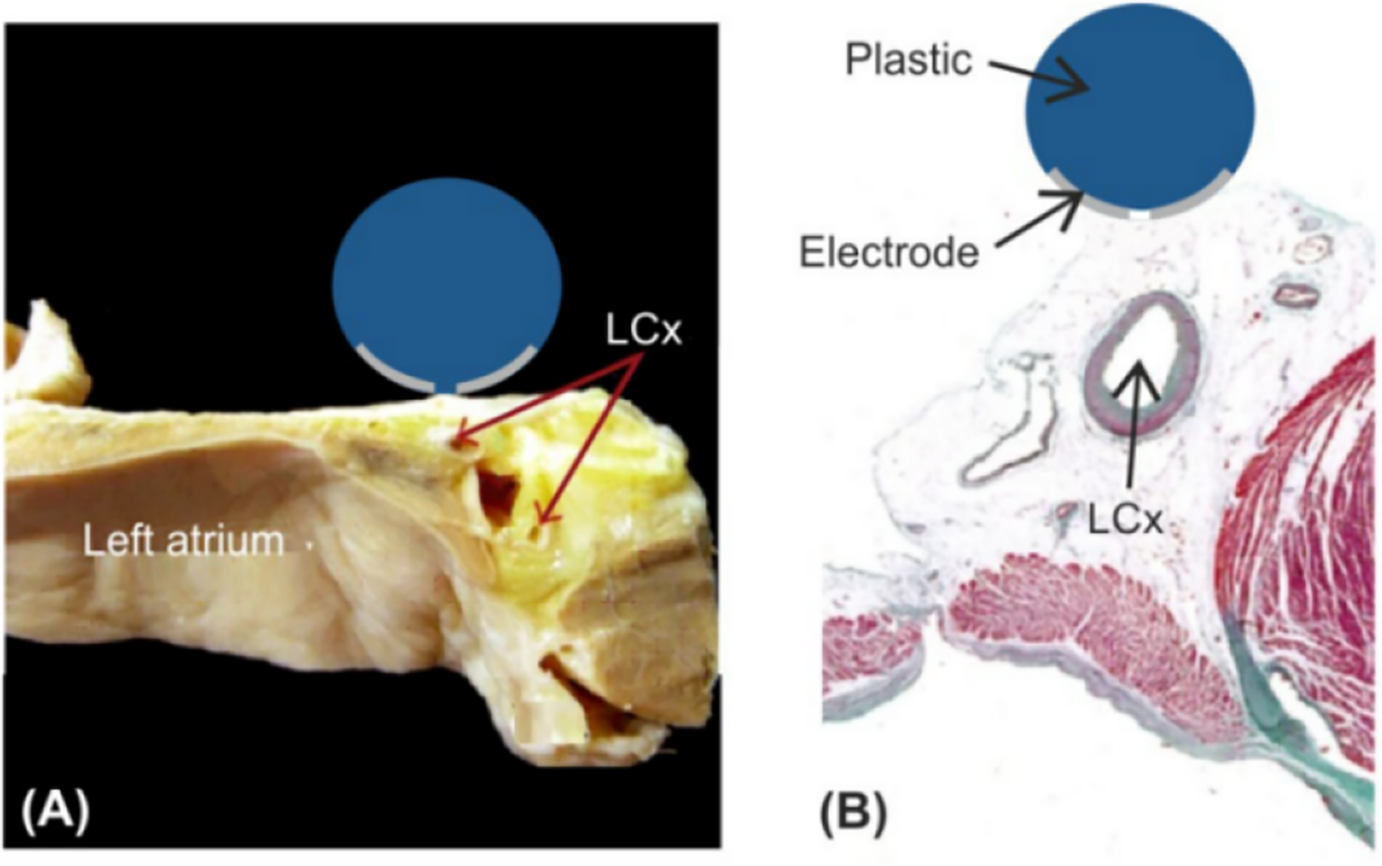
Schematic diagram of left atrial decortication (26, 29). Figure **(A)** Anatomical picture; Figure **(B)** Relation between the AE and the left coronary artery (LCx).

**Figure 2:**
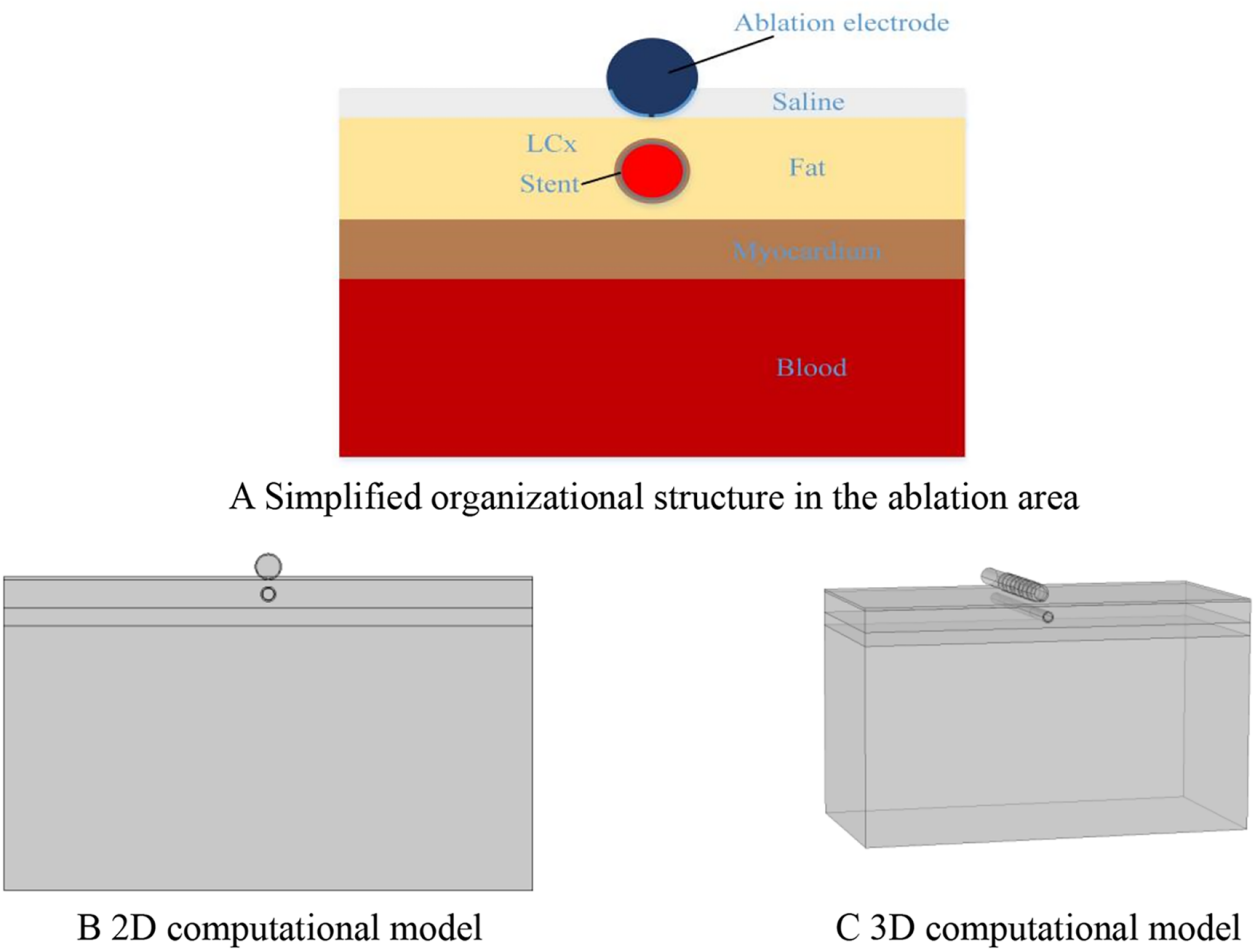
Computer simulation model.

To facilitate the comparison of the ablation effects of the two models, we only consider the ablation device and the surrounding area of interest when building the model, and this modeling method has been proven to be effective in previous studies (23–25). According to previous studies and the need for model simplification, we simplified the different tissues in the ablation area, modeled the ablation region of interest in layers (saline, fat, myocardium, and blood), and placed the ablation device above the fat layer according to the real structure of the human organ, in which the ablation device dimensions were set to realistically simulate the size of the ablation area, and the dimensions were identical to the real size of the ablation device. The size of the ablation device is the same as the real size of the ablation device, and the model is established as shown in Figure 1 (20, 23, 26). The PFA ablation position is shown in Figure 1, with the left coronary artery usually located directly below the ablation electrode. The ablation electrode has a diameter of 3.98 mm, a length of 2.54 mm, a 0.76 mm flushing hole in the center, and 0.5 mm saline on the upper side of the epicardial fat, which is used to act as a virtual electrode, and the ablation electrode and the fat are completely close to each other, which ensures that the ablation energy can be effectively transmitted to the ablation area (25–27). The computer simulation model is shown in Figure 2. The fat thickness in the model was 4.3 mm, the myocardial thickness was 2.7 mm, and the blood thickness and width models were set to X = 80 and Y = 40, respectively, concerning previous studies (23, 28).

### 2.2 Boundary conditions and pulse parameters

**Figure 3:**
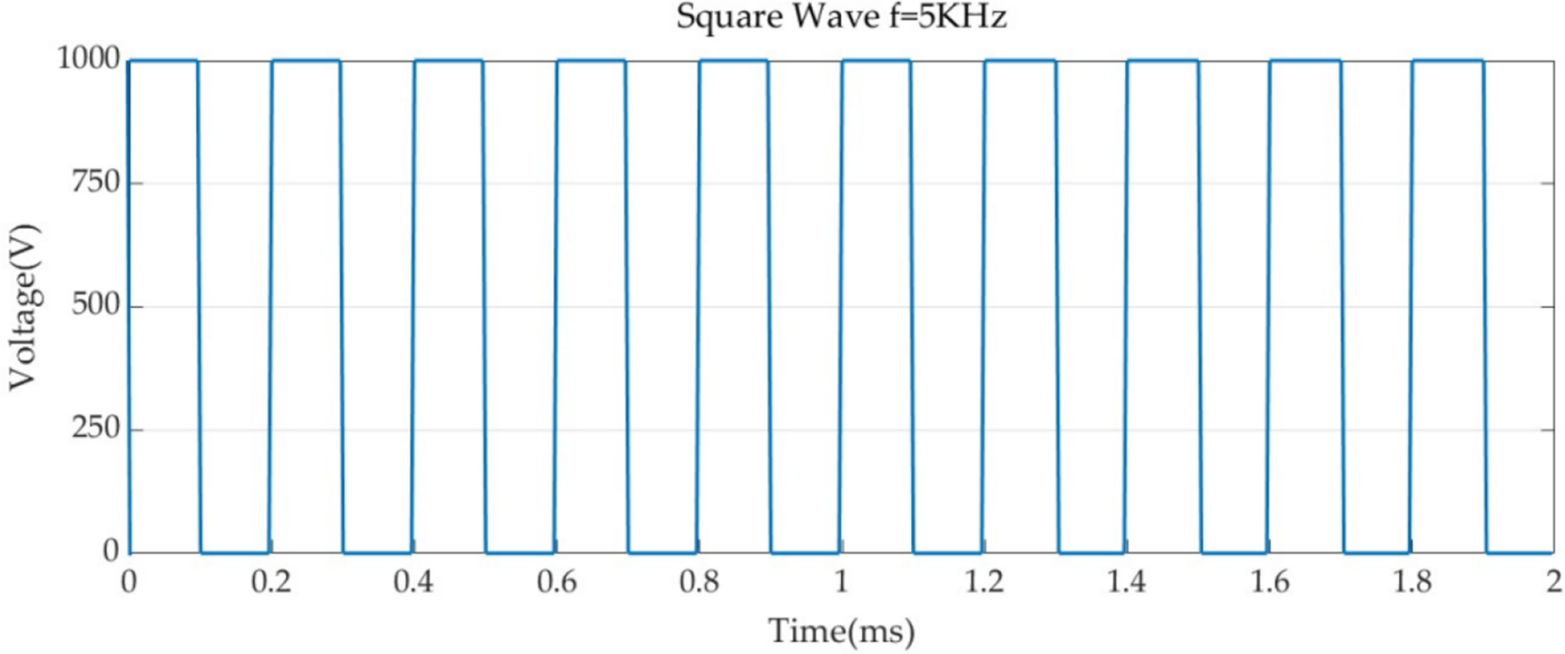
Pulse parameters.

Boundary conditions were set for each of the two simulation models, and 1,000 V was set for the PFA catheter ablation metal electrode with a pulse duration of 100 us and a pulse interval of 100 us (23, 30). The voltage pulse parameters are shown in Figure 3. For the metal electrode spacing insulator set 0 V, and set the voltage to 0 V for all the boundaries of the model, to show that the simulation process is not subject to external interference, and the electric field has the metal electrode spreading outward (25, 31).

To evaluate the effect of ablating different metal stents on temperature, we set thermal boundary conditions, for which thermal convection coefficients were applied at the air-electrode interface (20 W/m^2^ K, ambient temperature 21°C) and at the myocardium-blood interface to simulate blood circulation (1,417 W/m^2^ K, 37°C blood temperature) under high blood flow (24.4 cm/s) conditions (32–34). This is because blood flow occurs inside the coronary arteries due to the contraction of the heart chambers, so in the simulation, we took into account the characteristics of blood flow, and the cooling effect of tissues such as the heart chambers on the ablation area, we set the myocardial temperature to 37°C in the model. In the absence of a stent, we simplified the situation by assuming a flow rate of 0.5 m/s, half of the peak reported in, corresponding to a convection coefficient of 63.19 /m^2^ K calculated in (34, 35).

### 2.3 Material properties

The conductivity of different biological tissues varies from person to person, and appropriate values of biological properties can improve the calculation accuracy of computer simulation models. After long-term exploration by researchers, the conductivity of biological tissues shows an S-shaped distribution with the growth of voltage. This may be because the electric field applied by PFA changes the permeability of biological tissues, resulting in the gap in the cell membrane of biological tissues becoming larger when receiving the stimulation of the electric field, and the permeability of the electric current is enhanced resulting. In the paper, we also considered the change in conductivity of saline, blood, and other tissues with temperature so that the conductivity increased by 2% with temperature (30, 36–38). In our experiments, we used the sigmoid function to simulate the conductivity change of biological tissues, Eq. The various parameters of electrical and thermal properties needed for the simulation are shown in Table 1 (36, 39, 40).

$$\sigma (E,T)=(\sigma_{0}+\frac{\sigma_{1}-\sigma_{0}}{1+10e^{-}\frac{\left(|E|-58,000\right)}{3000}})\cdot 1.02^{T-37}$$

where 0 and 1 are the conductivity before and after electroporation, respectively, and the values of 0 and 1 are related to the presence and size of pores in the cell membrane. Before the appearance of PFA, i.e., when the pores have not yet formed, the current flows only through the extracellular material, which is the same as when the tissue is subjected to low-frequency electrical excitation, and the cell membrane acts as an electrical insulator. Under practical conditions, it was maintained without significant change between 1 and 10 kHz. At higher pulse frequencies, the conductivity on the myocardium decreases due to pore formation resulting in current flowing not only extracellularly, but also through the cytoplasm through the pores, but the regional effect of PFA ablation is negligible in other pulse frequency conditions (23, 37).

**Table 1:** Parameters for model conductivity calculation.

Mlement	Stent	Electrode	Poyurethane	Saline	Myocardium	Blood	Fat
σ_0_ (S/m)	7.4e^6^	4.6e^6^	1e^−5^	1.392	0.0537	0.7	0.0377
σ_1_ (S/m)					0.0281	0.748	0.0438

### 2.4 Control equations

To simulate the PFA ablation phenomenon in a real scenario, similar to the previous research methods, we chose to build a finite element simulation model in the simulation software COMSOL, and the geometry and finite element mesh used in the study are shown above, in which the catheter electrodes are located in the saline layer, which is used to simulate the real environment of the human body ablation. The various physical parameters required for the control equations are shown in Table 2. In the computational model, we added the biological heat transfer module, so the model belongs to the electro-thermal coupling problem, and in the current module, we introduced Laplace's system of equations to simulate the simulated electric field (30, 41, 42).

∇⋅(σ∇V)=0

**Table 2:** Control equations and parameters for bioheat calculations.

Mlement	Stent	Electrode	Poyurethane	Saline	Myocardium	Blood	Fat
k (W/m·K)	15	71	23	0.628	0.56	0.52	0.21
ρ (kg/m^3^)	8,000	21,500	1,440	980	1,081	1,050	911
c (J/kg·k)	480	132	1,050	4,184	3,686	3,617	2,348

E=−∇V

J=σE

where is the conductivity, the exact value of which is given above, *V* is the voltage, *E* is the electric field strength, and *J* is the current density.

For considering the heat generated during the PFA ablation process, we used the bioheat equation to investigate any possible side effects due to the heat source during the ablation process.

$$\rho c_{p}\frac{dT}{dt}=\nabla \cdot (k\nabla T)+Q+Q_{e}+Q_{met}$$

$$Q=\sigma |E{|}^{2}$$

Where ρ is the density in (kg/m^3^); *c* is the specific heat in (J/kg·K); T is the temperature in (°C); t is the time in (s); *k* is the thermal conductivity of the tissue in (W/m·K); *Q* is the heat source generated by the electric field, which is related to the field strength and conductivity in (W/m^3^); *Q_p_* is the heat loss due to blood flow in (W/m^3^); and *Q_met_* is the heat loss due to the biological metabolism in (W/m^3^).

### 2.5 Analysis of results

PFA ablation was simulated using finite element simulation to assess whether the presence of a metal stent during epicardial ablation affects the electric field distribution during ablation and also to simulate the change in temperature of the ablation region during ablation. In the paper, we simulated different scenarios: the position and orientation between the arterial stent and the ablation electrode, the distance between the ablation electrode and the metal stent, and the position of the different saline layers about the ablation electrode. We utilized the 1,000 V/cm contour to assess the maximum width and depth of the PFA ablation area, due to a study that recently reported an electric field threshold of 1,000 V/cm for PFA-induced irreversible myocardial damage (43). Temperature distributions were calculated to assess possible adverse thermal side effects, especially in the vicinity of ablation electrodes and metal stents.

## 3 Results

### 3.1 Electric field distribution

**Figure 4:**
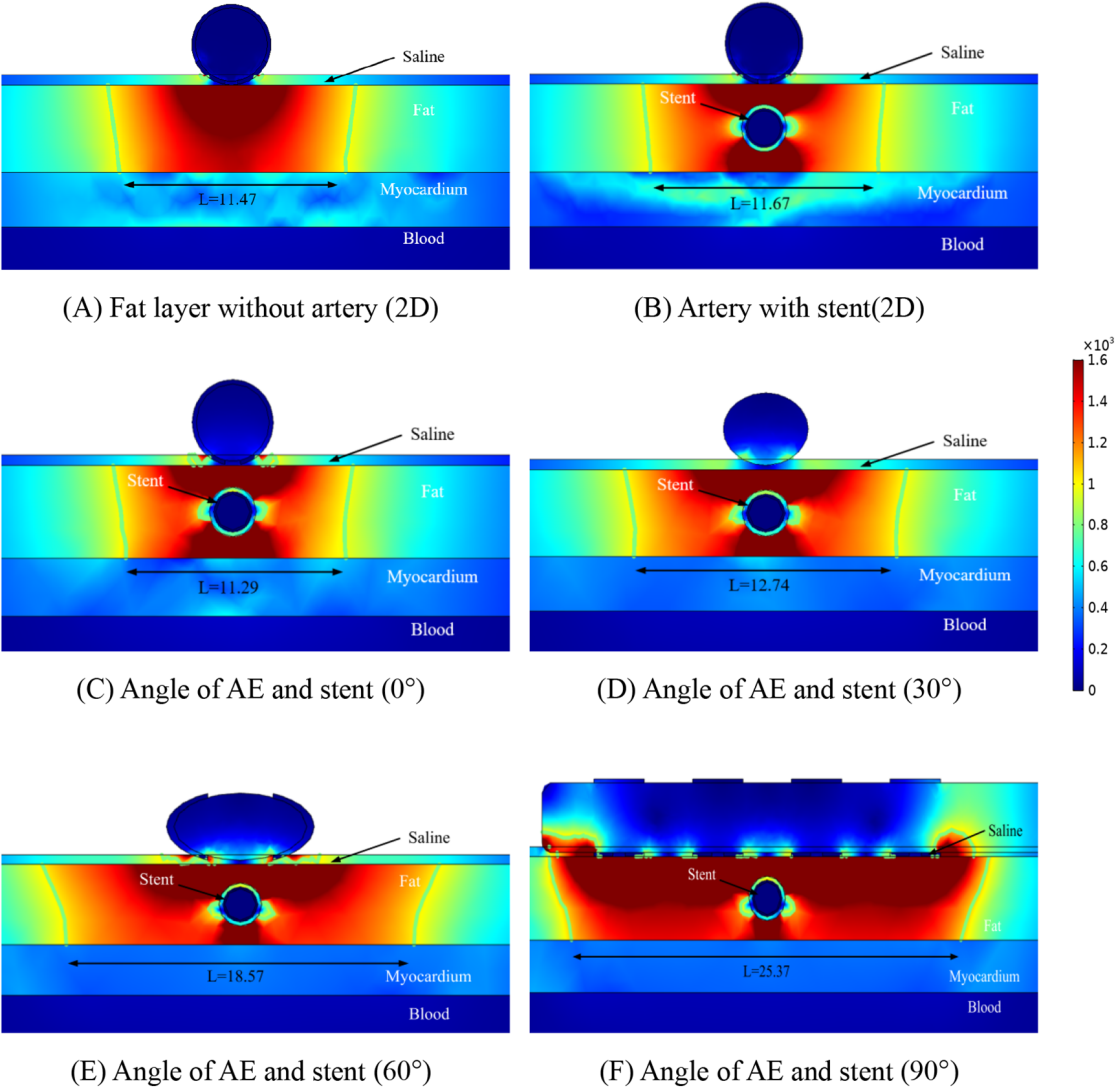
Schematic diagram of the electric field distribution in the ablation area of the catheter and stent at different positions of 1 mm, with an ablation voltage of 1,000 V, and the number of ablation pulses of 10, with an interval of 0.1 ms between each pulse. Figures **(A,B)** show 2D simulations of epicardial pulsed fields; Figures **(C–F)** show 3D simulations of epicardial pulsed fields.

The distribution of the electric field in the ablation region is shown in Figure 4 for different conditions at four positions. Our prerequisite was to perform ablation simulation experiments under the condition of ten pulses utilizing the ablation catheter (pulse duration of 0.1 ms and pulse interval of 0.1 ms). In the ablation experiments, we set the coronary arteries to contain a metal stent inside, and at the same time, the distance between the metal stent and the ablation catheter was 1 mm. It can be seen from Figure 4 that the presence of the metal stent inside the coronary arteries affects the normal distribution state of the electric field. This finding is similar to the results of previous studies, while the angular relationship between the coronary artery and the ablation catheter affects the distribution of the electric field around the artery, and it can be concluded from Figure 4 that when the direct angle between the ablation catheter and the coronary artery is 0°, the distribution of the electric field will be higher on both sides of the upper and lower sides of the coronary artery. At the same time, this phenomenon also occurs between different angles of the coronary artery and the ablation catheter, and this finding can also be derived from the electric field distributions of the simulated regions at different positional angles in Figure 4.

Due to the presence of the coronary arteries, an abnormal distortion of the electric field distribution occurs during PFA ablation, but it can be concluded from Figure 4 that this distorted electric field exists only inside the fat layer and has no effect on the cardiomyocytes. In Figure 4, we labeled the ablation regions at different angles, and inside the green wireframe is the region where the electric field strength is greater than 1,000/cm, and it is derived from the literature that this region may cause damage to cardiomyocytes. We measured the width and height of different angles to the ablation catheter in the axial direction of the coronary arteries, and we found that the width of the ablation changes as the angle between the coronary arteries and the metal stent changes, but these ablation areas do not penetrate the fat layer. The width of the ablation area was 11.47 mm in Figure 4A, 11.67 mm in Figure 4B, 11.29 mm in Figure 4C, 12.74 mm in Figure 4D, 18.57 mm in Figure 4E, and 25.37 mm in Figure 4F.

As shown in Figure 4, in the ablation cross-section, we can see that the region with electric field strength greater than 1,000/cm is located in the fat layer, which does not affect the myocardial layer cells, and we find that in the axial cross-section, the region with stronger electric field distribution is the position of the left and right sides of the coronary artery, and the electric field distribution in this region is distorted, which suggests that the presence of the coronary artery and the metal stent affects the distribution of the normal electric field, but this distortion of the electric field is confined to the fat layer. As shown in Figures 4C–F, for the axial position of the coronary artery, the electric field generated by the ablation electrodes at different angles showed an angular difference with the coronary artery, and the distortion of the electric field by the coronary artery still existed, and the location and direction of the electric field distortion and the angular direction of the ablation electrodes had an effect.

**Figure 5:**
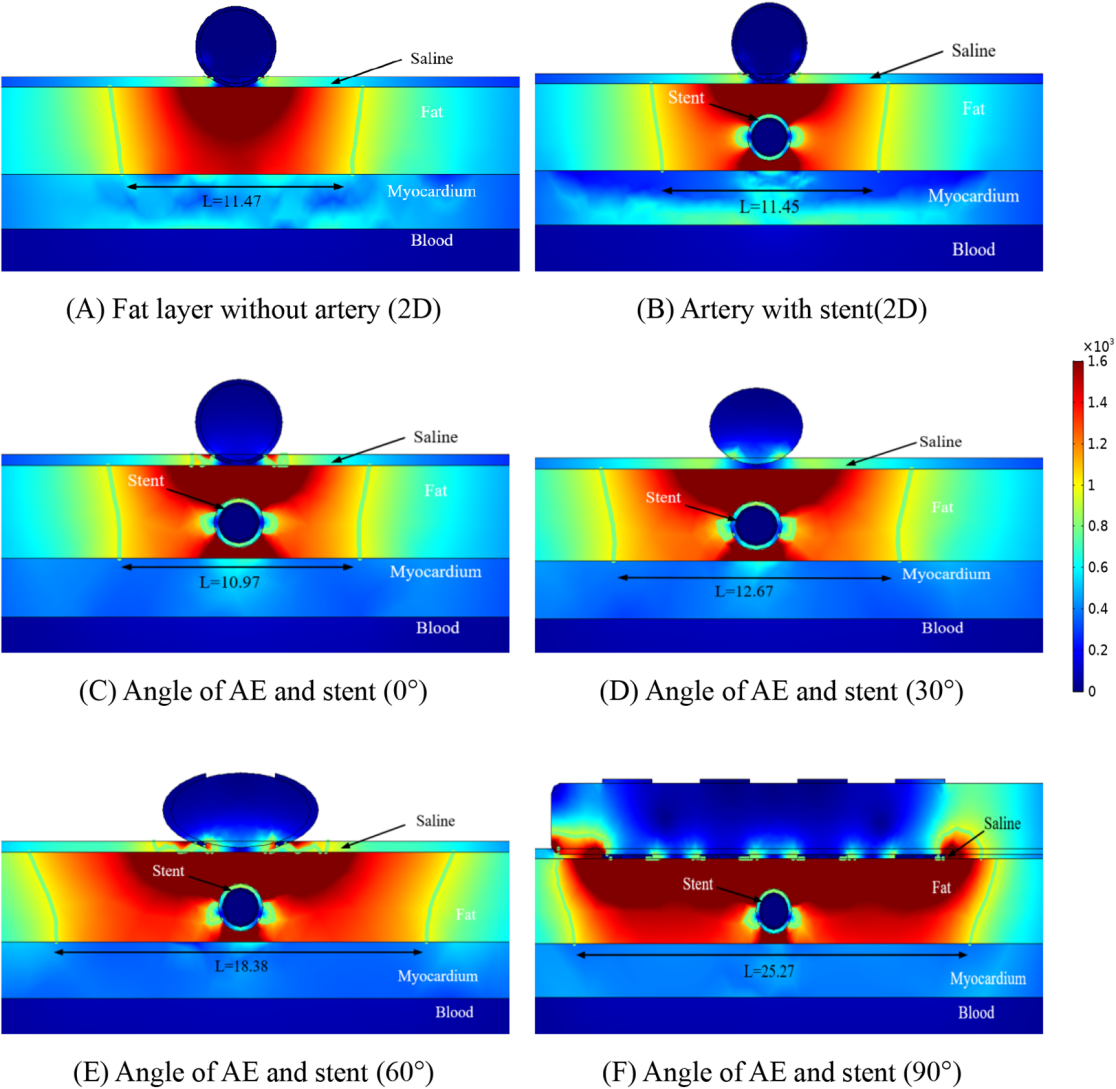
Schematic diagram of the electric field distribution in the ablation area of the catheter and stent at different positions of 1.5 mm, with an ablation voltage of 1,000 V, and the number of ablation pulses of 10, with an interval of 0.1 ms between each pulse. Figures **(A,B)** show 2D simulations of epicardial pulsed fields; Figures **(C–F)** show 3D simulations of epicardial pulsed fields.

From Figure 4, it can be obtained that the AE generates the ablation electric field only in the fat layer, and there is no damage to the myocardium under the fat layer. Figure 5 shows the electric field distribution in the axial direction of the CMS at different angles between the ablation catheter and the coronary artery at a distance of 15 mm, and it can be obtained from Figure 5 that the distorting influence of the coronary artery on the distribution of the electric field still exists, and the width of the ablation region is 11.47 mm in Figure 5A, 11.45 mm in Figure 5B, 10.97 mm in Figure 5C, 12.67 mm in Figure 5D, 18.38 mm in Figure 4E, and 25.27 mm in Figure 4F.

Similar to Figure 4, the focus of electric field distribution is still on the left and right sides of the coronary arteries, and the distribution of electric field distortion is correlated with the position of the ablation electrode, which is similar to that shown in Figure 4. Analysis of Figures 4, 5 showed that the distance between the metal stent and the ablation electrode had a weak effect on the width of the ablation, which decreased when the distance between the two increased, and we also found that the change in the position of the metal stent did not affect the depth of ablation, which was limited to the fat layer in our study. The intensity of the electric field inside the coronary arteries was much less than the threshold for myocardial injury (1,000 v/cm), which suggests that the electric field did not produce a threshold injury inside the coronary arteries.

### 3.2 Temperature distribution

**Figure 6:**
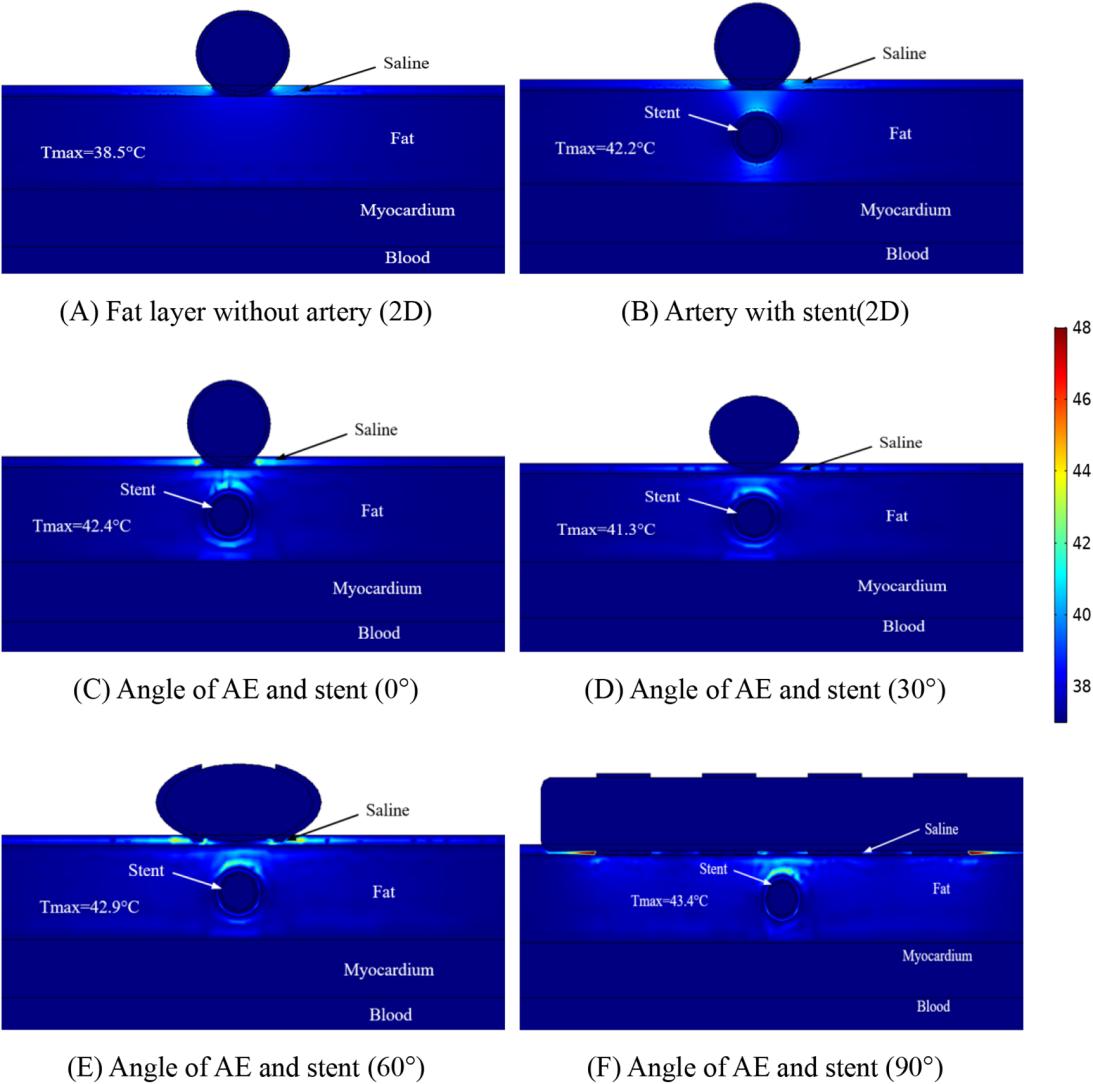
Schematic diagram of the electric field distribution in the ablation area of the catheter and stent at different positions of 1 mm, with an ablation voltage of 1,000 V, and the number of ablation pulses of 10, with an interval of 0.1 ms between each pulse. Figures **(A,B)** show 2D simulations of epicardial pulsed fields; Figures **(C–F)** show 3D simulations of epicardial pulsed fields.

The temperature distribution of the ablation region was another focus of our study, and we performed temperature measurements for different positions of the ablation region to obtain the maximum temperature region at different positions. Figure 6 shows the maximum temperature distribution in the ablation region when the ablation catheter was 10 mm from the stromal artery. From Figure 6, it can be obtained that the presence of the metal stent and the coronary artery increases the maximum temperature in the ablation region, while the angle between the ablation electrode and the metal stent effects the temperature in the ablation region because of the presence of the ablation electrode and the metal stent. In Figure 6A the maximum temperature of the ablation region is 38.5 °C, in Figure 6B the maximum temperature of the ablation region is 42.2 °C, in Figure 6C the maximum temperature of the ablation region is 42.4 °C, in Figure 6D the maximum temperature of the ablation region is 41.3 °C, in Figure 6E the maximum temperature of the ablation region is 42.9 °C and in Figure 6F the maximum temperature of the ablation region is 43.4 °C. As shown in Figure 6, we also obtain that for different computer simulation models, the maximum temperature in the ablation region is almost unchanged (42.2°C for the 2D model and 42.4°C for the 3D model), and the temperature difference between the two is only 0.2°C. In our study, we found that the maximum temperature in the ablation area was 43.4°C when the angle between the metal stent and the ablation electrode was 90°. From this, we can conclude that the different positions of the ablation catheter do not have much effect on the temperature inside the ablation area, and the small temperature change will not cause discomfort to the clinical patients due to the change of temperature, it also provides meaningful simulation data to prove that pulsed-field ablation of atrial fibrillation can be performed for post-stenting patients.

**Figure 7:**
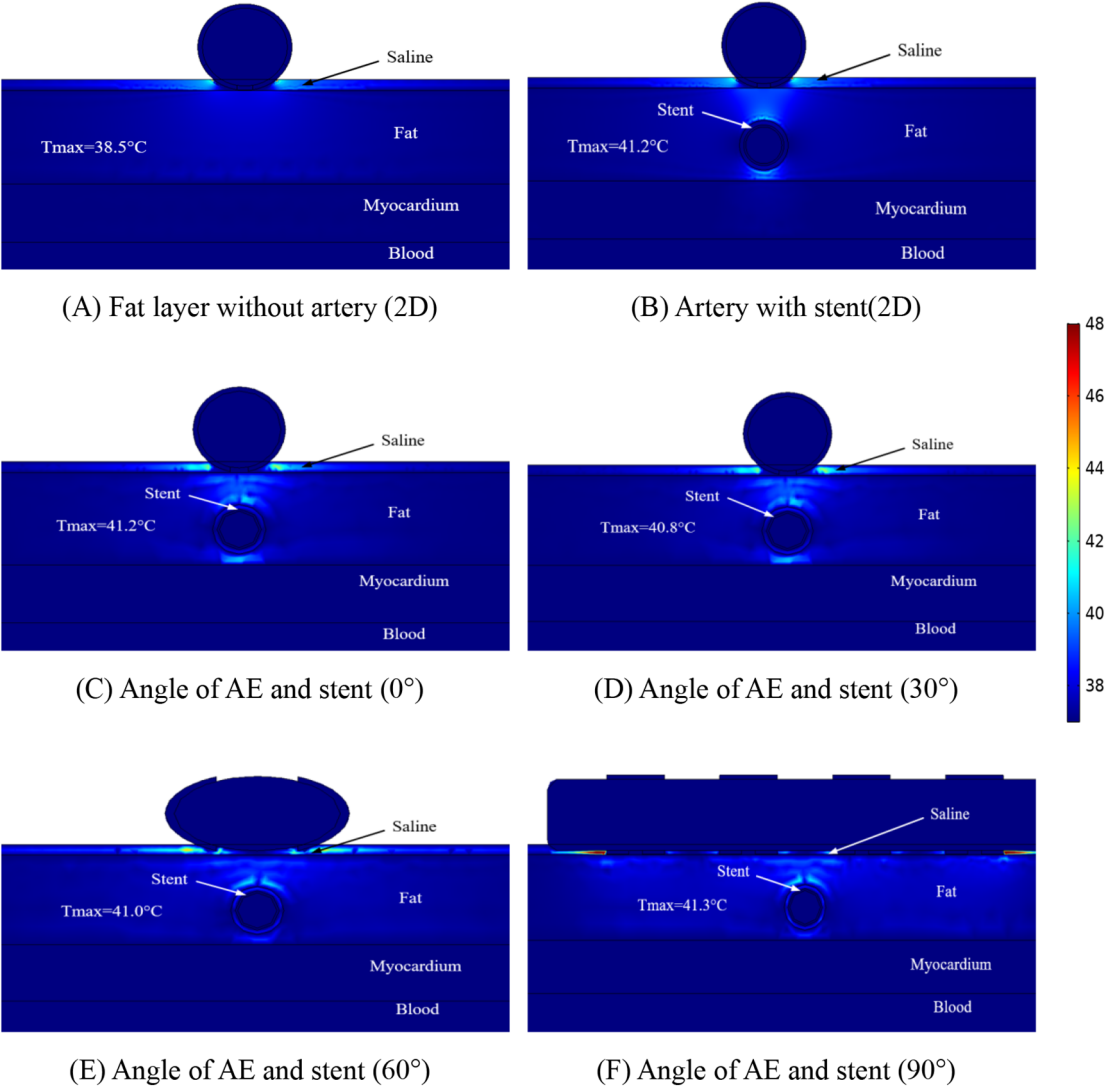
Schematic diagram of the electric field distribution in the ablation area of the catheter and stent at different positions of 1 mm, with an ablation voltage of 1,000 V, and the number of ablation pulses of 10, with an interval of 0.1 ms between each pulse. Figures **(A,B)** show 2D simulations of epicardial pulsed fields; Figures **(C–F)** show 3D simulations of epicardial pulsed fields.

To study the temperature variation of the ablation region in depth, we made simulations for different distances between the coronary artery and the ablation catheter, which is because this situation may exist in different patients, and the distributions of the temperature when the distance between the coronary artery and the ablation catheter was 1.5 mm are shown in Figure 7. The maximum temperature in the ablation region in Figure 7A is 38.5°C; the maximum temperature in the ablation region in Figure 7B is 41.2°C; the maximum temperature in the ablation region in Figure 7C is 41.2°C; the maximum temperature in the ablation region in Figure 7D is 40.8°C; the maximum temperature in the ablation region in Figure 7E is 41.0°C; and the maximum temperature in the ablation region in Figure 7F is 41.3°C.

Figure 7 shows that the maximum temperature in the ablation region is 41.3°C and the minimum temperature in the ablation region is 38.5°C. We also found that the temperature in the ablation region changes when the angular position of the ablation electrode and the metal bracket changes, and the maximum temperature in the ablation region is 40.8°C when the angle between the two is 30° in this study. In this study, the maximum temperature in the ablation region was 40.8°C at an angle of 30°. The maximum temperature in the ablation region was 41.3°C at an angle of 90°. The change of angle has less effect on the temperature of the ablation region (maximum temperature difference is 0.5°C), and this result is consistent with that shown in Figure 6, in which the maximum temperature difference in the ablation region is 1.2°C.

## 4 Discussion

### 4.1 Findings

At present, the safety and efficacy of PFA in the treatment of AF are still worth exploring, and a large number of studies have proved that endocardial ablation can effectively reduce the chance of damaging the tissues of the esophagus, but there are fewer studies on epicardial ablation regarding tissue damage (44, 45). Recently, it has been demonstrated that due to the presence of metal stents inside the coronary arteries, the ablation process in organisms can cause an increase in temperature in the local area, which may be due to the conductive nature of the metal (21, 46). It has also been demonstrated that the presence of coronary arteries as well as metal stents affects the normal electric field and temperature distribution acted by the ablation catheter (17, 23, 27). However, to date, we are not aware of any study that has evaluated the effect of metal stents inside the coronary arteries on the electric field and temperature during epicardial ablation from different perspectives, and to the best of our knowledge, our study is the first to utilize computational modeling to evaluate the effect of the coronary arteries and metal stents on the distribution of the electric field and the distribution of the temperature during the epicardial ablation process from different perspectives. In our study, three-dimensional modeling was performed using computers to simulate the spatial position of the ablation catheter and coronary arteries. The main results of this study are as follows:

1. The presence of coronary arteries with metallic stents distorts the normal electric field distribution in the ablation region, and this again appears to be consistent in the three-dimensional and two-dimensional models.
2. There is an effect of the angle of the ablation electrode to the metal bracket on the width of the ablation area, but there is no change in the position of the cold spots of the electric field present on both sides of the metal bracket (left and right sides of the metal bracket).
3. Comparing different calculation models, the ablation area of the three-dimensional model is wider than the ablation area of the two-dimensional model, with a difference of 0.38 mm in the ablation area width when the distance between the ablation electrode and the metal bracket is 10 mm, and a difference of 0.46 mm in the ablation area width when the distance is 10 mm.
4. There is a temperature increase between the ablation electrode and the metal support, and the highest point of temperature distribution is near the ablation electrode, with the highest temperature in the ablation area differing by 1.2℃ when the distance between the ablation electrode and the metal support is 10 mm, and the highest temperature in the ablation area differing by 0.1℃ when the distance is 15 mm.
5. Different angles between the ablation catheter and the coronary artery affect the distribution of temperature in the ablation area, with the highest temperature in the ablation area decreasing and then increasing as the angle changes, independent of the distance between the two.
6. The presence of the metal support has a distorting effect on the electric and temperature fields, and this distortion is present in the fat layer region.

We determined the validity of the three-dimensional modeling by comparing the data from the two-dimensional computer model with the three-dimensional computer model. In our computational model, the ablation region of the 2D computational model was wider than that of the 3D computational model (approximately 0.4 mm), which may be attributed to the small difference in organ thickness and model meshing that we considered when modeling the 3D model. Our results are similar to those of previous studies in that the ablation area was mainly distributed near the ablation electrodes, a phenomenon that does not change due to the distance between the ablation electrodes and the coronary arteries (see Figures 4, 5).

We found that the width of the ablation area changes with the distance between the ablation electrode and the coronary artery, and the width of the ablation area increases by 0.32 mm when the distance between the ablation electrode and the coronary artery is increased by 0.5 mm, and this phenomenon does not change due to a change in the angle between the two (see Figures 4, 5). A comparison of Figures 4, 5 reveals that the distance between the coronary artery and the myocardial layer does not result in damage to the myocardial layer from the electric field (0.5 mm decrease in the distance between the coronary artery and the myocardial layer).

For the first time, we assessed the temperature within the ablation region using a three-dimensional model, and in our model, the distance between the ablation catheter and the coronary artery effected the temperature in the ablation region, with the temperature in the ablation region decreasing by 2.1℃ (in terms of the maximum temperature) when the distance between the two was increased, but these temperature changes did not cause damage to other tissues. The temperature in the ablation region is shown in Figures 6, 7 to be the smallest when the angle between the coronary artery and the ablation catheter is 30°, which may be becausethe cross-section we took did not contain an ablation electrode, and the temperature may increase in other ablation regions, but this increase in temperature does not exceed the temperatures shown in Figures 6C, 7C.

Finally, there is now a large body of research demonstrating the safety and efficacy of proving PFA in endocardial treatment of AF, including the fact that it can be effective in reducing the chance of complications such as phrenic nerve injury. In this study of ours, ablation treatment of AF from the epicardium using PFA means that the ablation catheter is closer to the metal stent, which would make the ablation energy to the target area of ablation more affected by the metal stent, and catheter ablation near the metal stent was safe in our simulations. This may mean that the ablation effect is less likely to be affected by the metal stent during endocardial ablation and validates the safety and reliability of endocardial PFA ablation.

### 4.2 Limitations

In our 3D computational model, the cross-section for measuring electric field and temperature was the radial cross-section of the coronary artery and the metal stent, which was to facilitate the comparison of the electric field distribution and temperature distribution on the same cross-section in different models, and we did not compare the data on other cross-section, which might result in the incomplete acquisition of the ablation data in the model, resulting in the under-analysis of the pulsed ablation characteristics. Nevertheless, we analyzed the electric field and temperature distributions at different angles between the ablation electrode and the coronary artery at the same cross-section in different models, and we also analyzed the two extreme conditions during epicardial ablation, namely, the extreme conditions of 0° and 90° angle between the ablation electrode and the coronary artery, and therefore we believe that our calculations have relevant clinical significance from a physical point of view.

## 5 Conclusion

This paper compares the simulation data of the 3D finite domain model and 2D finite domain model. 1. The consistency of the simulation data between the 2D finite domain model and 3D finite domain model is obtained, which provides data reference for the simplified model establishment afterwards. 2. The width of the ablation area is investigated by using 3D computational model, and the positional relationship between the coronary artery and the ablation catheter is found to effect the width of the ablation area. 3. The temperature of the ablation region was investigated using a three-dimensional computational model, and the highest temperature in the ablation region was obtained at the extreme condition of a 90° angle between the ablation electrode and the coronary artery. This may be due to the presence of the metal stent in the ablation area causing a normal distribution of the electric field, and this distortion of the electric field raises the temperature in the ablation area (less than 44°C, with the maximum temperature between the ablation electrode and the coronary artery). However, this increase in temperature does not produce thermal damage to the corresponding tissues.

## Data Availability

The original contributions presented in the study are included in the article/Supplementary material, further inquiries can be directed to the corresponding authors.
